# Supplementary material for: Estimation of cell lineages in tumors from spatial transcriptomics data
Source: Nat Commun. 2023 Feb 2;14:568. doi: 10.1038/s41467-023-36062-6 (PMC9895078; doi:10.1038/s41467-023-36062-6)
Supplement: Supplementary file 1 — Supplementary Information [file 41467_2023_36062_MOESM1_ESM.pdf]

Supplementary Information

This PDF file includes:
Supplementary Figures 1-18
Supplementary Tables 1-4

Supplementary Figures

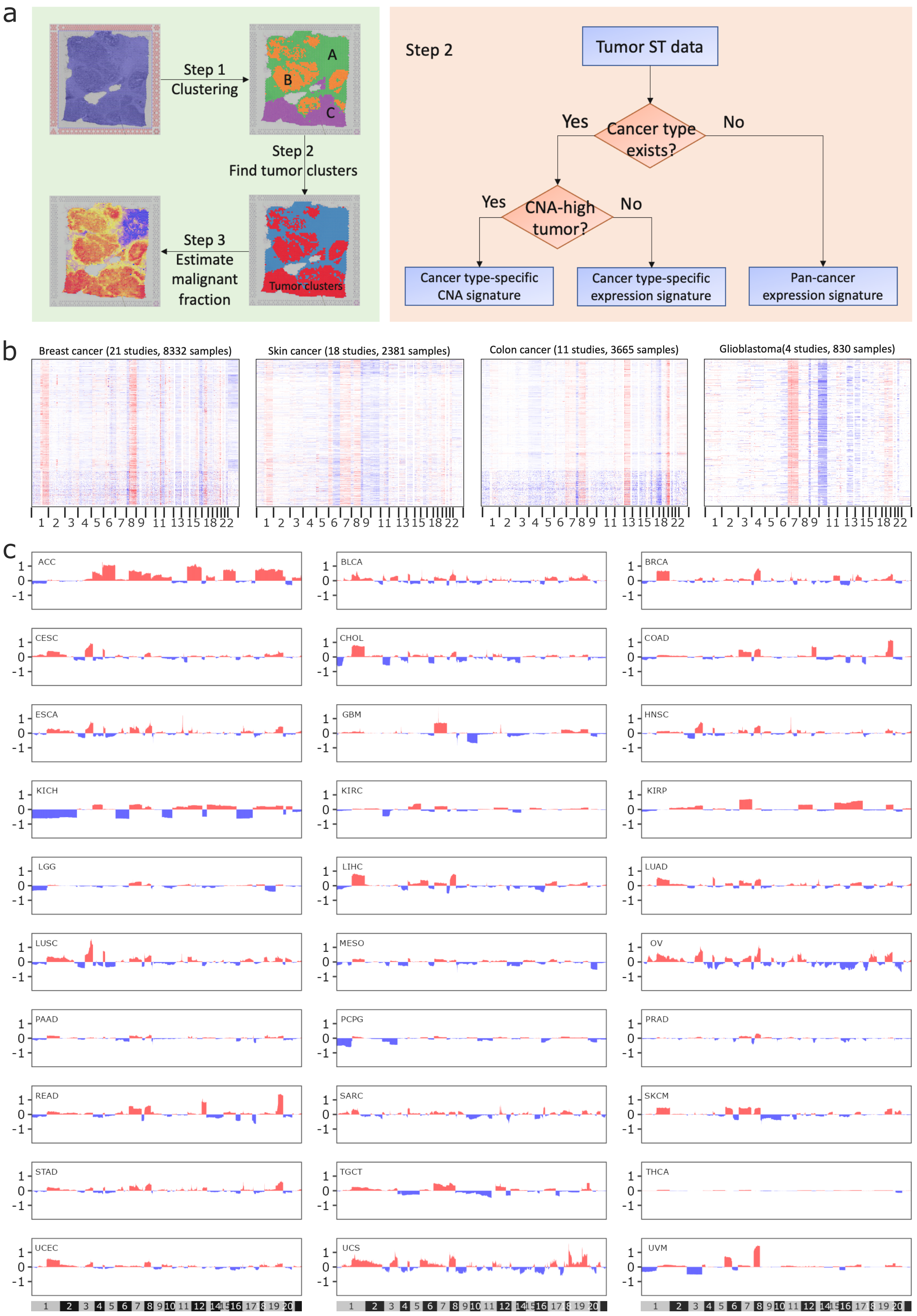

Supplementary Fig. 1. Inference of malignant cell fraction, related to Fig. 1

a, *Three steps to infer malignant cell fraction without any reference, based on a dictionary of cancer type-specific gene patterns.* 1) Clustering all spots from a tumor ST dataset by hierarchical clustering. 2) Determining the malignant cell clusters whose spots have significant correlations with cancer type-specific patterns, with pattern selection rules shown in the right panel. 3) Estimating malignant cell abundance across all spots. For a tumor ST dataset, the ST-specific malignant expression profile was computed as the average expression profile among spots from malignant cell clusters in step 2. Then, the expression profile of each spot within a tumor ST dataset was correlated to the ST-specific malignant profile to infer the malignant cell fraction.

b, *Copy number alteration (CNA) pattern across various data sets for the same cancer type in cBioPortal database.* Red, amplifications; blue, deletions.

c, *Cancer type-specific CNA dictionary for TCGA cancer types*, computed as the average CNA values (y-axis) over chromosomal locations (x-axis) across patients. Each vertical bar represents a gene, and all genes are sorted along chromosomes.

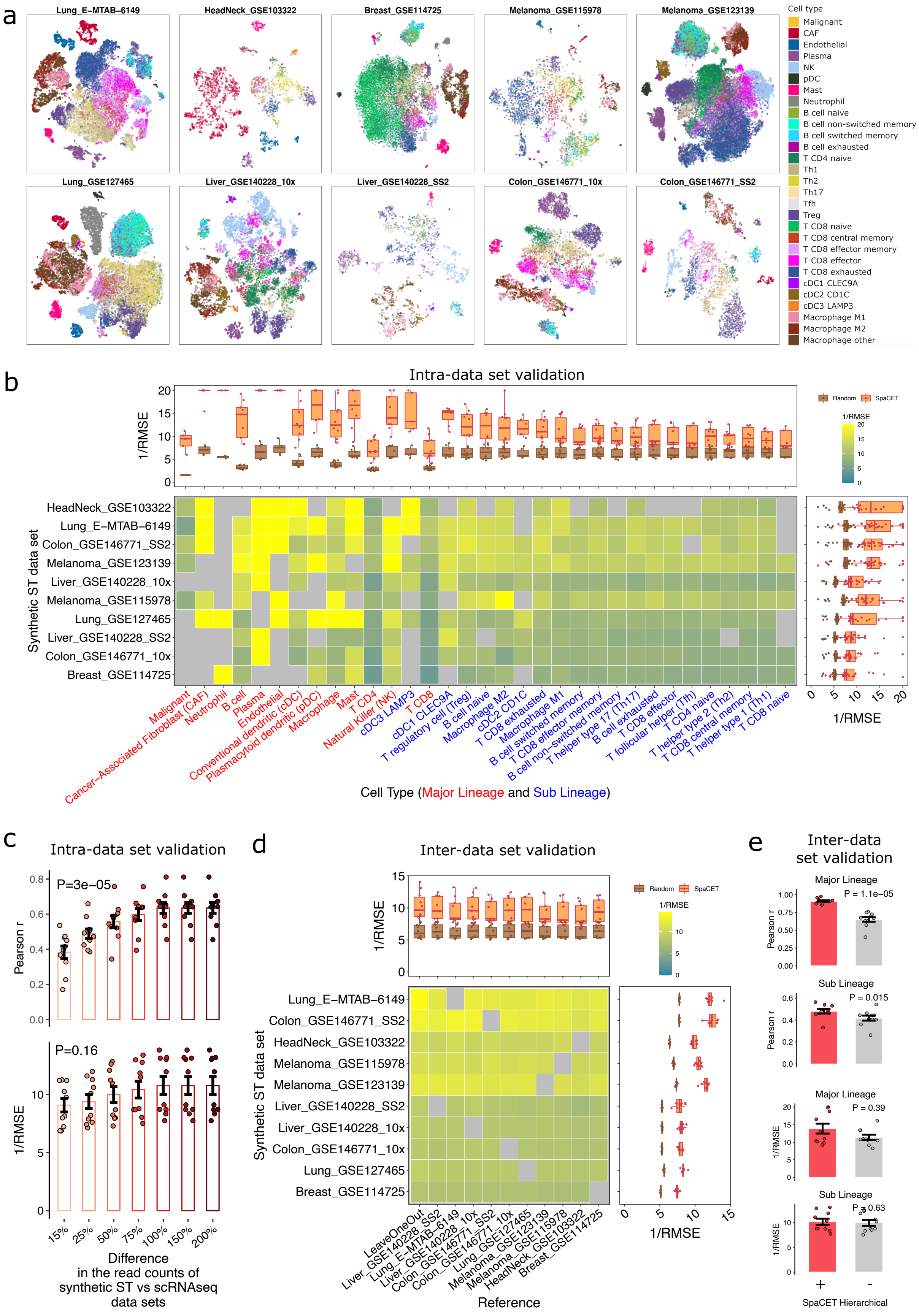

a
Lung_E−MTAB−6149
HeadNeck_GSE103322
Breast_GSE114725
Melanoma_GSE115978
Melanoma_GSE123139
Lung_GSE127465
Liver_GSE140228_10x
Liver_GSE140228_SS2
Colon_GSE146771_10x
Colon_GSE146771_SS2
Cell type
Malignant
CAF
Endothelial
Plasma
NK
pDC
Mast
Neutrophil
B cell naive
B cell non−switched memory
B cell switched memory
B cell exhausted
T CD4 naive
Th1
Th2
Th17
Tfh
Treg
T CD8 naive
T CD8 central memory
T CD8 effector memory
T CD8 effector
T CD8 exhausted
cDC1 CLEC9A
cDC2 CD1C
cDC3 LAMP3
Macrophage M1
Macrophage M2
Macrophage other
b
Intra-data set validation
1/RMSE
Random
SpaCET
Synthetic ST data set
HeadNeck_GSE103322
Lung_E−MTAB−6149
Colon_GSE146771_SS2
Melanoma_GSE123139
Liver_GSE140228_10x
Melanoma_GSE115978
Lung_GSE127465
Liver_GSE140228_SS2
Colon_GSE146771_10x
Breast_GSE114725
Malignant
Cancer−Associated Fibroblast (CAF)
Neutrophil
B cell
Plasma
Endothelial
Conventional dendritic (cDC)
Plasmacytoid dendritic (pDC)
Macrophage
Mast
T CD4
Natural Killer (NK)
cDC3 LAMP3
T CD8
cDC1 CLEC9A
T regulatory cell (Treg)
B cell naive
Macrophage M2
cDC2 CD1C
T CD8 exhausted
Macrophage M1
B cell switched memory
T CD8 effector memory
B cell non−switched memory
T helper type 17 (Th17)
B cell exhausted
T CD8 effector
T follicular helper (Tfh)
T CD4 naive
T helper type 2 (Th2)
T CD8 central memory
T helper type 1 (Th1)
T CD8 naive
Cell Type (Major Lineage and Sub Lineage)
c
Intra-data set validation
P=3e−05
Pearson r
P=0.16
1/RMSE
15%
25%
50%
75%
100%
150%
200%
Difference
in the read counts of
synthetic ST vs scRNAseq
data sets
d
Inter-data set validation
1/RMSE
Random
SpaCET
Synthetic ST data set
Lung_E−MTAB−6149
Colon_GSE146771_SS2
HeadNeck_GSE103322
Melanoma_GSE115978
Melanoma_GSE123139
Liver_GSE140228_SS2
Liver_GSE140228_10x
Colon_GSE146771_10x
Lung_GSE127465
Breast_GSE114725
LeaveOneOut
Liver_GSE140228_SS2
Lung_E−MTAB−6149
Liver_GSE140228_10x
Colon_GSE146771_SS2
Colon_GSE146771_10x
Lung_GSE127465
Melanoma_GSE123139
Melanoma_GSE115978
HeadNeck_GSE103322
Breast_GSE114725
Reference
e
Inter-data
set validation
Major Lineage
P = 1.1e−05
Pearson r
Sub Lineage
P = 0.015
Major Lineage
P = 0.39
1/RMSE
Sub Lineage
P = 0.63
+
-
SpaCET Hierarchical

Supplementary Fig. 2. Performance validation of SpaCET by ST data simulation, related to Fig. 2

a, *t-SNE plot of ten single-cell RNA-seq (scRNA-seq) datasets.* Each dot presents a single cell. The t-distributed stochastic neighbor embedding (t-SNE) was used to project the *scRNA-seq* profiles in two dimensions with distances between dots representing the profile similarities.

b, *Performance in intra-dataset validation for each scRNA-seq dataset (row) and cell type (column)*. The color in the heatmap presents 1/root mean square error (RMSE) between predicted vs known cell fractions. The gray color in the heatmap indicates missing cell types in the scRNA-seq dataset. Box plots on the top present 1/RMSE values of the same cell type across all scRNA-seq datasets (n=10). Box plots on the right present 1/RMSE values of all cell type predictions in the same scRNA-seq dataset. Across spots of each synthetic ST data, we shuffled spot identities of cell type fraction vectors as random controls. The thick line represents the median value. The bottom and top of the boxes are the 25th and 75th percentiles (interquartile range). The whiskers encompass 1.5 times the interquartile range.

c, *Impact of the read count differences of ST vs scRNA-seq data.* Each dot represents a simulated ST dataset synthesized from a single scRNA-seq dataset (n=10). The y axis presents the median Pearson correlation r and RMSE between predicted and known cell fractions across all cell types. The difference of groups was evaluated by the Kruskal-Wallis test. Bar height denotes average value across simulated ST datasets; error bars denote standard errors.

d, *Performance of inter-dataset validation between scRNA-seq cohorts*. The labels in the column and row axis show the scRNA-seq data sets (n=10) used to generate cell type reference profiles and synthetic ST data, respectively. The color in the heatmap represents the median 1/RMSE between predicted and known cell fractions across all cell types. The gray color in the heatmap indicates that the same dataset was not used to generate both reference profiles and synthetic ST data. Box plots on the top show median 1/RMSE values for all synthetic ST data decomposed by the same reference profile. Box plots on the right show median 1/RMSE values for the same synthetic ST data decomposed by all reference profiles. All boxplots with random controls are plotted as panel b.

e, *Deconvolution results of SpaCET with (red bar) and without (gray bar) hierarchical lineage***.** A dot represents a simulated ST dataset synthesized from a single scRNA-seq dataset (n=10). Each simulated ST dataset was decomposed by using a leave-one-out signature, which is the reference derived from all scRNA-seq datasets except the one left out to synthesize the simulated ST data. The y-axis presents the median Pearson correlation r and 1/RMSE between predicted and known cell fractions across cell types. The difference of groups was evaluated by the two-sided Wilcoxon rank sum test. Bar height denotes average value across simulated ST datasets; error bars denote standard errors.

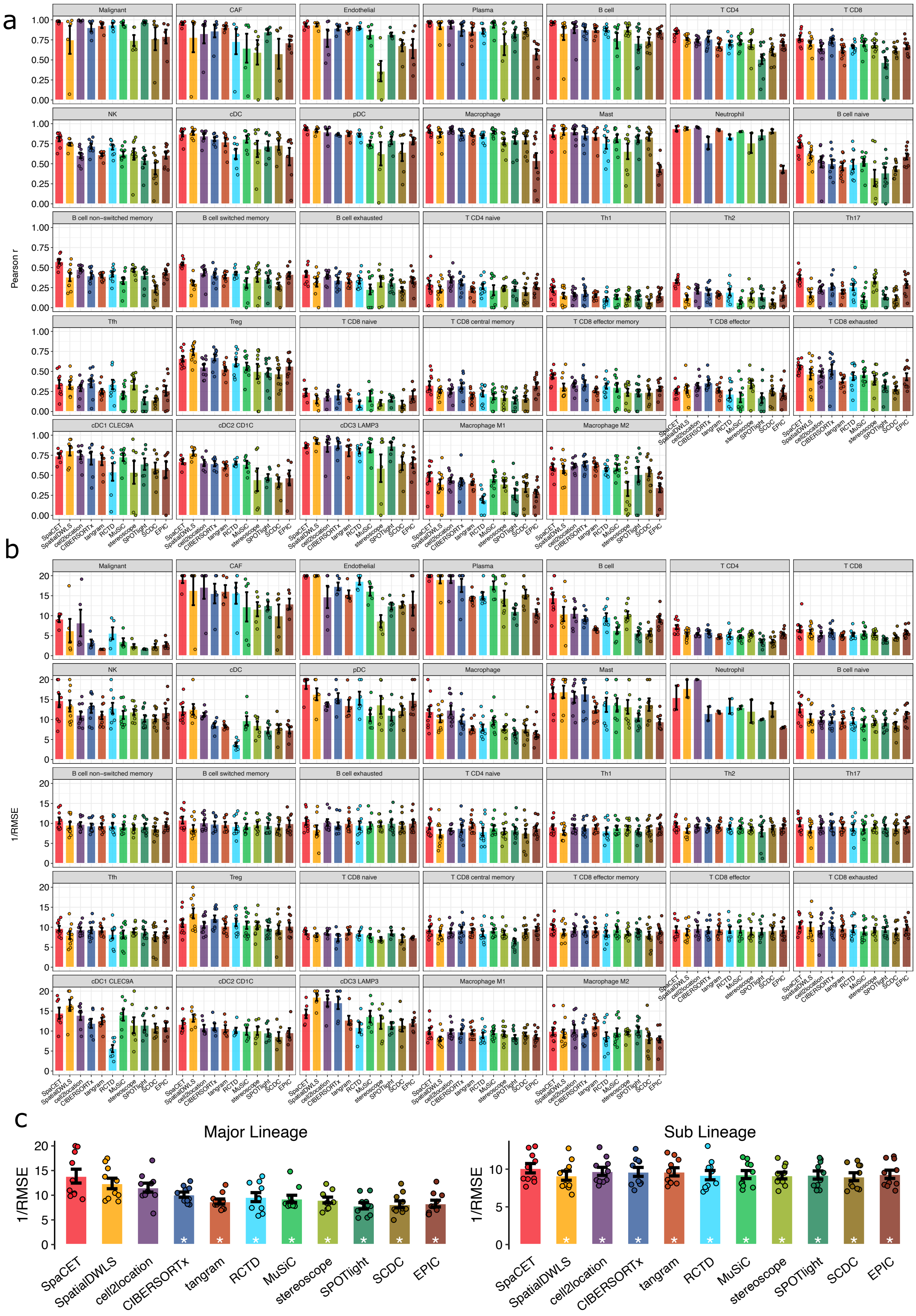

a
Malignant
CAF
Endothelial
Plasma
B cell
T CD4
T CD8
NK
cDC
pDC
Macrophage
Mast
Neutrophil
B cell naive
B cell non-switched memory
B cell switched memory
B cell exhausted
T CD4 naive
Th1
Th2
Th17
Tfh
Treg
T CD8 naive
T CD8 central memory
T CD8 effector memory
T CD8 effector
T CD8 exhausted
cDC1 CLEC9A
cDC2 CD1C
cDC3 LAMP3
Macrophage M1
Macrophage M2
Pearson r
0.00
0.25
0.50
0.75
1.00
SpaCET
SpatialDWLS
cell2location
CIBERSORTx
tangram
RCTD
MuSiC
stereoscope
SPOTlight
SCDC
EPIC
b
1/RMSE
0
5
10
15
20
c
Major Lineage
Sub Lineage
1/RMSE

Supplementary Fig. 3. Performance comparisons by ST data simulation, related to Fig. 2.
a and b, *Performance comparison of inter-dataset validation for SpaCET and previous methods***.** A dot represents a simulated ST dataset synthesized from a single scRNA-seq dataset (n=10). The y-axis presents the Pearson correlation r (a) and 1/RMSE (b) between predicted and known cell fractions. All tools used the leave-one-out signature of 10 scRNA-seq datasets. Bar height denotes average value across simulated ST datasets; error bars denote standard errors. RMSE: Root Mean Square Error.
c, *Performance comparison summary.* A dot represents a simulated ST dataset synthesized from a single scRNA-seq dataset (n=10). The y-axis presents the median 1/RMSE between predicted and known cell fractions across cell types. All tools used the leave-one-out signature of ten scRNA-seq datasets. The difference between SpaCET and other tools was evaluated by the two-sided Wilcoxon signed-rank test. Bar height denotes average value across simulated ST datasets; error bars denote standard errors.

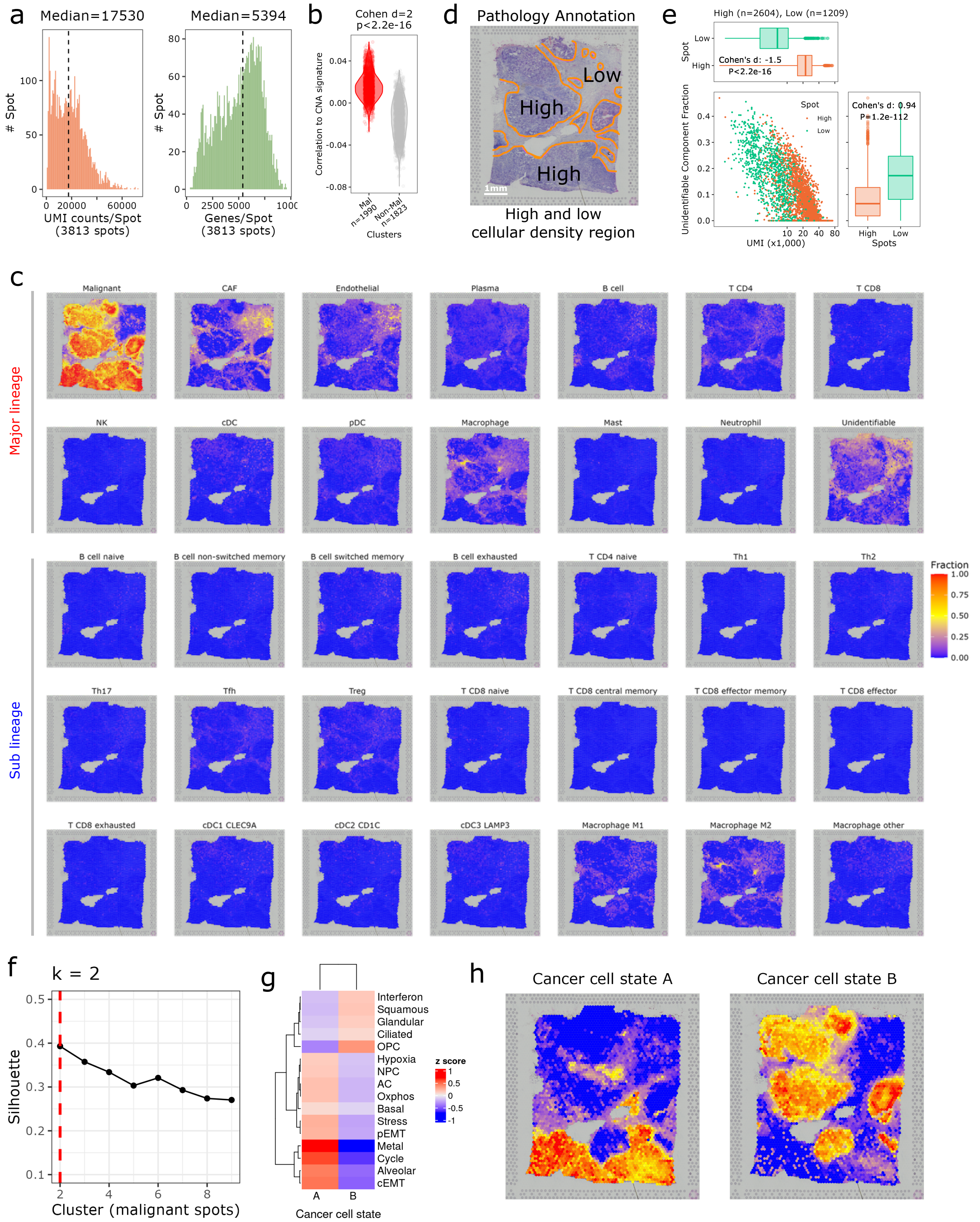

Supplementary Fig. 4. **SpaCET results of fresh-frozen breast tumor ST data**, related to Fig. 3

a, *Histogram of Unique Molecular Identifier (UMI) and gene counts per spot.* The dashed line represents the median value across all spots.

b, *Pearson correlation between the cancer type-specific CNA signature and the gene expression of spots in both malignant and non-malignant clusters.* A dot represents a spot. Two groups were compared by calculating the Cohen's d effect size and two-sided Wilcoxon rank-sum test. Each group is also summarized as a violin plot smoothed by a kernel density function.
c, *Spatial distribution of cell fractions predicted by SpaCET.*
d, *H&E image with pathology annotations of high and low cellular density regions.*
e, *The association between unidentifiable components and UMI counts across ST spots.* All spots were grouped in the high (n=2604) and low (n=1209) cellular density regions. The values of two groups were compared by using Cohen's d effect size and two-sided Wilcoxon rank-sum test. For the boxplot, the thick line represents the median value. The bottom and top of the boxes are the 25th and 75th percentiles (interquartile range). The whiskers encompass 1.5 times the interquartile range.
f, *Clustering and silhouette analysis of malignant spots to identify cancer cell states.* The point preceding the largest decrease in silhouette value was selected as the optimal cluster number.
g, *The scores of sixteen cancer gene modules for two cancer cell states.* The score of a cancer gene module for a malignant cell spot is the average expression level of all genes in this module. The score of a module for a cancer cell state is the average score of all spots belonging to this cancer cell state.
h, *Spatial distribution of two cancer cell states.*

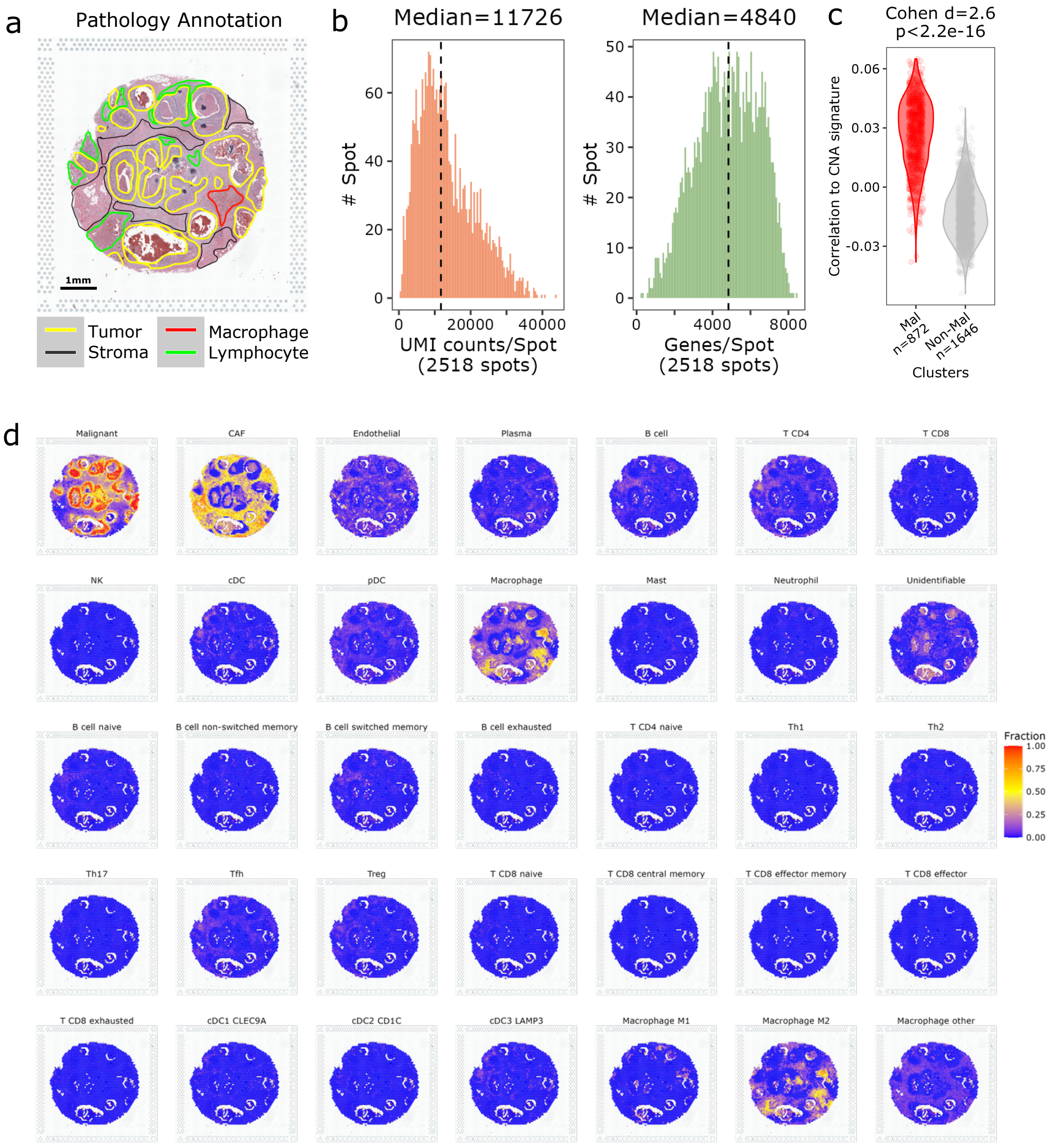

Supplementary Fig. 5. **SpaCET results of FFPE breast tumor ST data**, related to Fig. 3

a, *The H&E image with pathology annotations.*

b, *Histogram of UMI counts and genes per spot.* The dashed line represents the median value across all spots.

c, *Pearson correlation between the cancer type-specific CNA signature and the gene expression of spots in both malignant and non-malignant clusters.* A dot represents a spot. Two groups were compared by calculating the Cohen's d effect size and two-sided Wilcoxon rank-sum test. Each group is also summarized as a violin plot smoothed by a kernel density function.

d, *Spatial distribution of various cell types across the tumor tissue.*

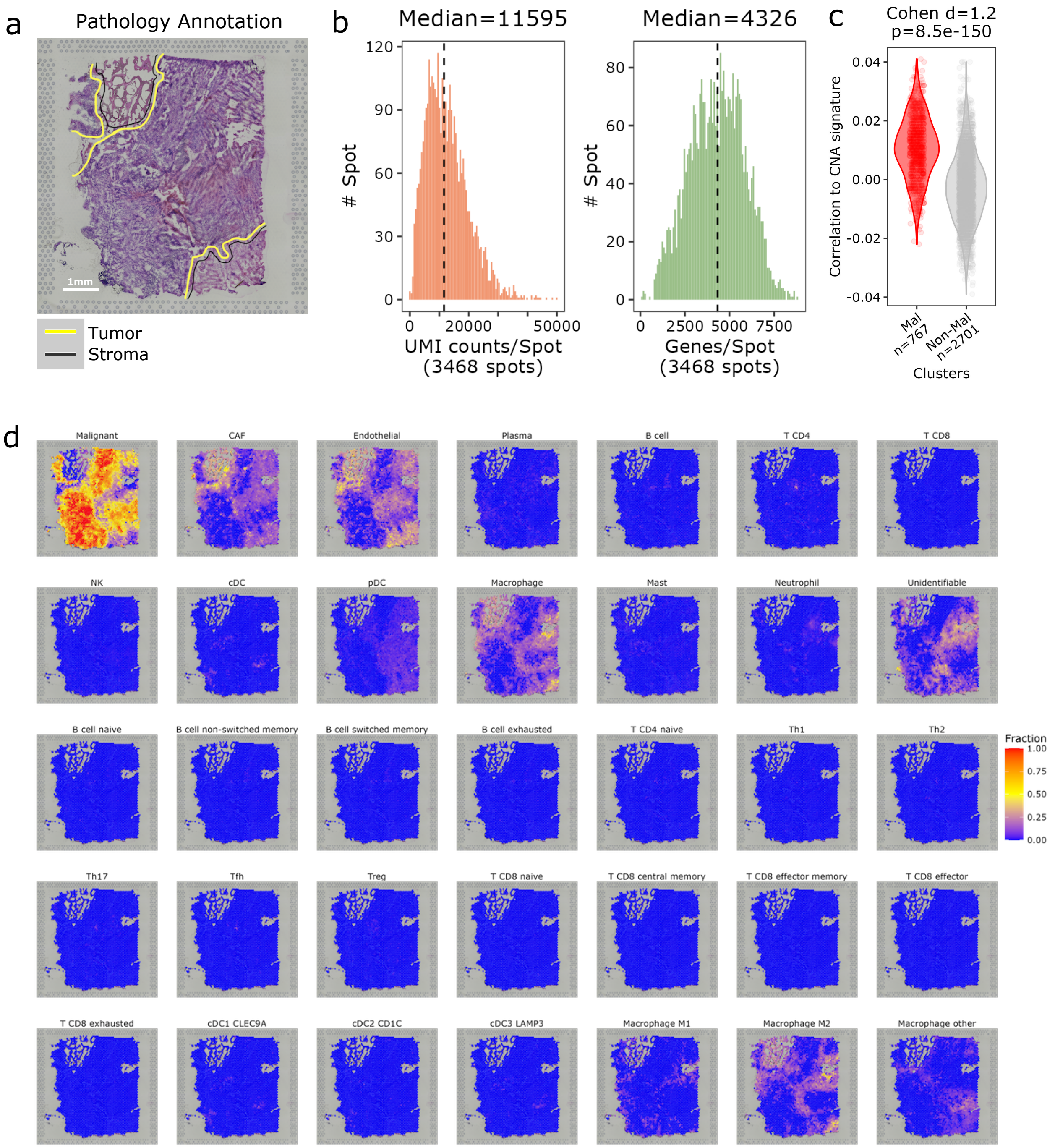

Supplementary Fig. 6. **SpaCET results of Glioblastoma ST data**, related to Fig. 3

a, *The H&E image with pathology annotations.*

b, *Histogram of UMI counts and genes per spot.* The dashed line represents the median value across all spots.

c, *Pearson correlation between the cancer type-specific CNA signature and the gene expression of spots in both malignant and non-malignant clusters.* A dot represents a spot. Two groups were compared by calculating the Cohen's d effect size and two-sided Wilcoxon rank-sum test. Each group is also summarized as a violin plot smoothed by a kernel density function.

d, *Spatial distribution of various cell types across the tumor tissue*.

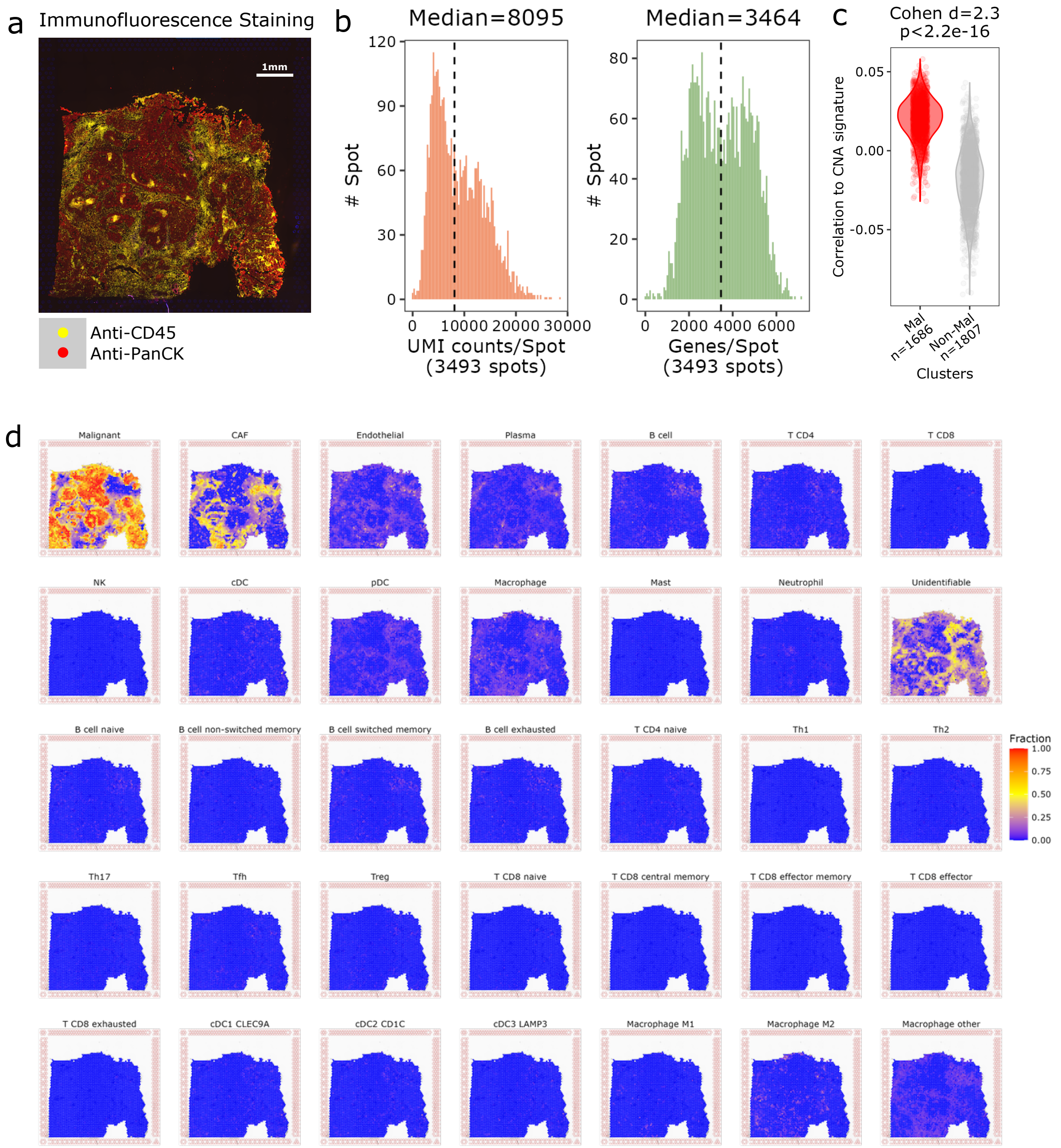

Supplementary Fig. 7. **SpaCET results of Ovarian tumor ST data**, related to Fig. 3

a, *Immunofluorescence staining image of cancer Pan-CytoKeratin (CK) and immune CD45 regions.* For CD45 cells, we included B, T CD4, T CD8, NK cells, and macrophages.

b, *Histogram of UMI counts and genes per spot.* The dashed line represents the median value across all spots.

c, *Pearson correlation between the cancer type-specific CNA signature and the gene expression of spots in both malignant and non-malignant clusters.* A dot represents a spot. Two groups were compared by calculating the Cohen's d effect size and two-sided Wilcoxon rank-sum test. Each group is also summarized as a violin plot smoothed by a kernel density function.

d, *Spatial distribution of various cell types across the tumor tissue.*

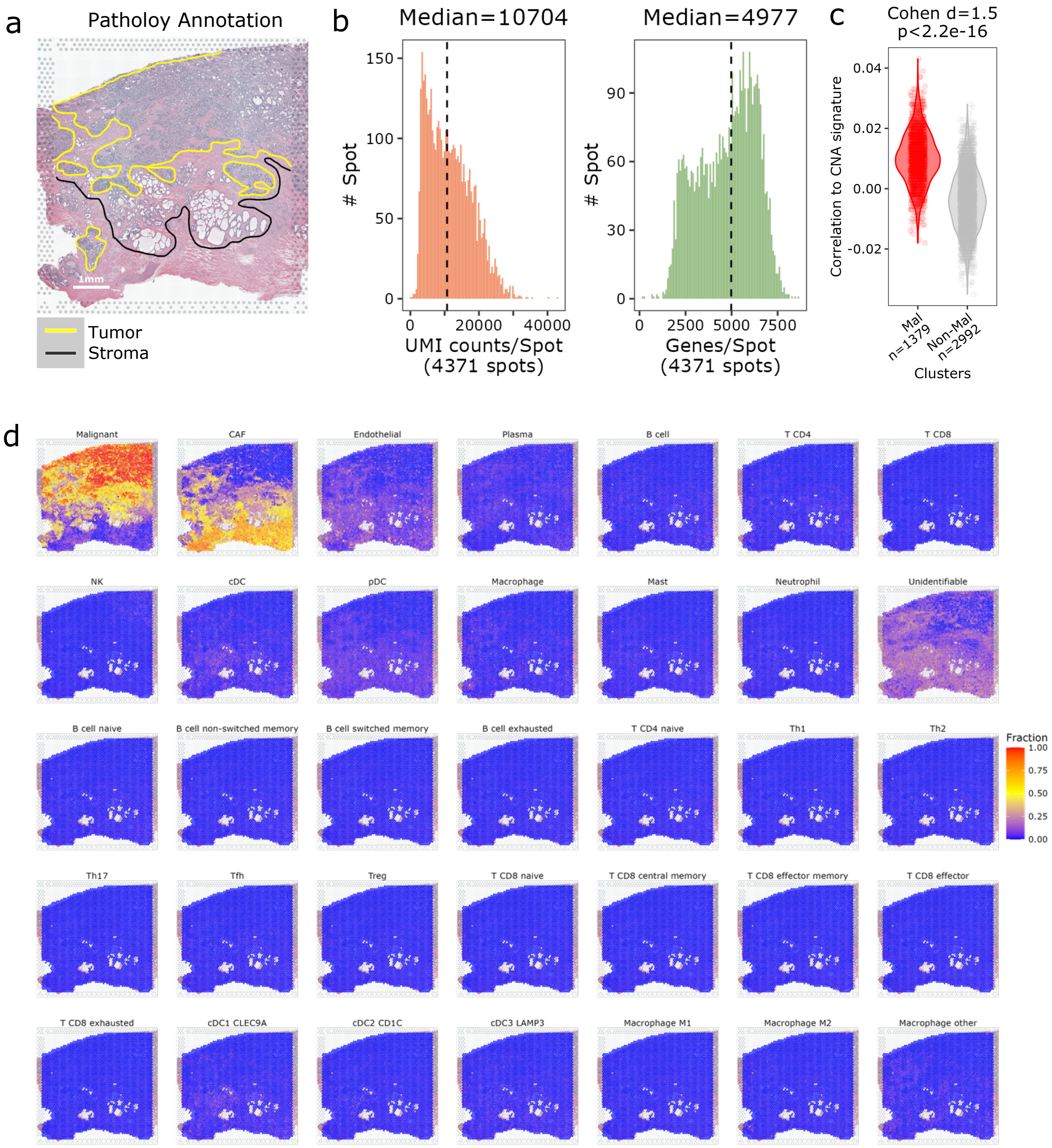

Supplementary Fig. 8. **SpaCET results of Prostate tumor ST data**, related to Fig. 3

a, *The H&E image with pathology annotations.*

b, *Histogram of UMI counts and genes per spot.* The dashed line represents the median value across all spots.

c, *Pearson correlation between the cancer type-specific CNA signature and the gene expression of spots in both malignant and non-malignant clusters.* A dot represents a spot. Two groups were compared by calculating the Cohen's d effect size and two-sided Wilcoxon rank-sum test. Each group is also summarized as a violin plot smoothed by a kernel density function.

d, *Spatial distribution of various cell types across the tumor tissue.*

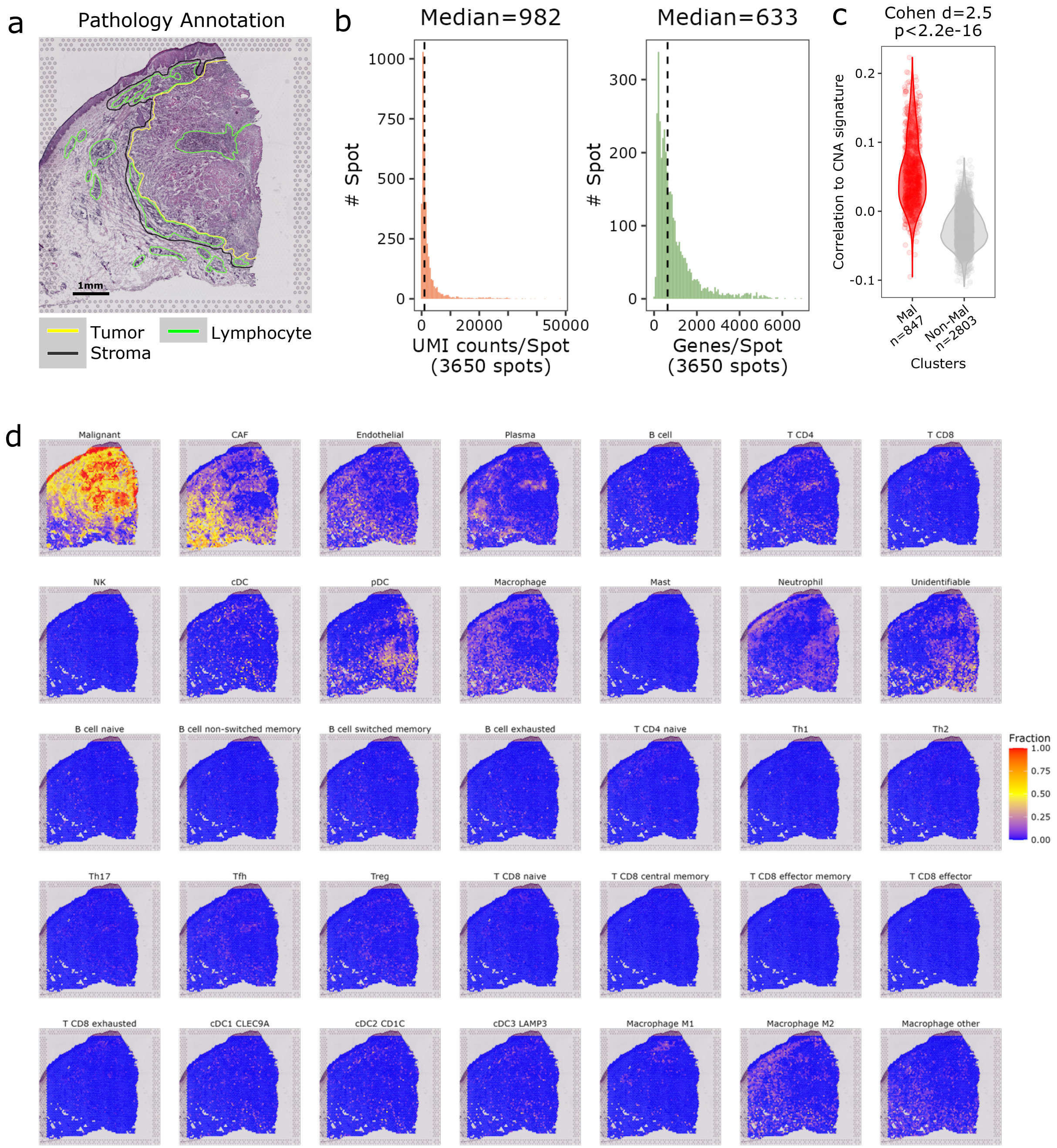

Supplementary Fig. 9. **SpaCET results of squamous cell carcinoma ST data**, related to Fig. 3

a, *The H&E image with pathology annotations.*

b, *Histogram of UMI counts and genes per spot.* The dashed line represents the median value across all spots.

c, *Pearson correlation between the cancer type-specific CNA signature and the gene expression of spots in both malignant and non-malignant clusters.* A dot represents a spot. Two groups were compared by calculating the Cohen's d effect size and two-sided Wilcoxon rank-sum test. Each group is also summarized as a violin plot smoothed by a kernel density function.

d, *Spatial distribution of various cell types across the tumor tissue*.

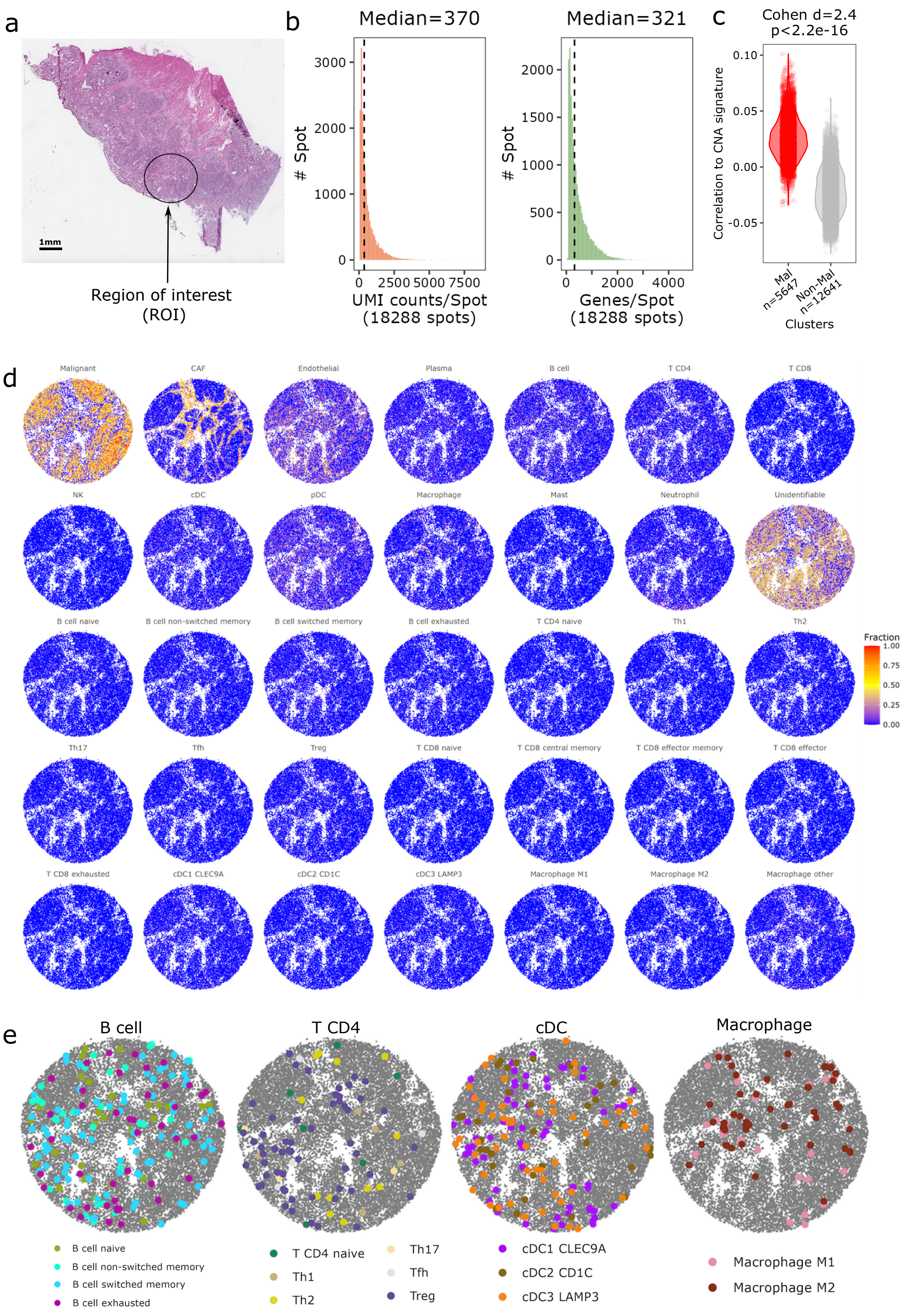
a
1mm
Region of interest
(ROI)
b
Median=370
# Spot
UMI counts/Spot
(18288 spots)
Median=321
# Spot
Genes/Spot
(18288 spots)
c
Cohen d=2.4
p<2.2e-16
Correlation to CNA signature
Mal
n=5647
Non-Mal
n=12641
Clusters
d
Malignant
CAF
Endothelial
Plasma
B cell
T CD4
T CD8
NK
cDC
pDC
Macrophage
Mast
Neutrophil
Unidentifiable
B cell naive
B cell non-switched memory
B cell switched memory
B cell exhausted
T CD4 naive
Th1
Th2
Fraction
Th17
Tfh
Treg
T CD8 naive
T CD8 central memory
T CD8 effector memory
T CD8 effector
T CD8 exhausted
cDC1 CLEC9A
cDC2 CD1C
cDC3 LAMP3
Macrophage M1
Macrophage M2
Macrophage other
e
B cell
T CD4
cDC
Macrophage
B cell naive
B cell non-switched memory
B cell switched memory
B cell exhausted
T CD4 naive
Th1
Th2
Th17
Tfh
Treg
cDC1 CLEC9A
cDC2 CD1C
cDC3 LAMP3
Macrophage M1
Macrophage M2

Supplementary Fig. 10. **SpaCET results of colon cancer slide-RNA-seq data**, related to Fig. 3 and Fig. 4

a, *The H&E image.* From a colon cancer tissue, a region of interest (ROI) was selected for the Slide-seq analysis.

b, *Histogram of UMI counts and genes per spot.* The dashed line represents the median value across all spots.

c, *Pearson correlation between the cancer type-specific CNA signature and the gene expression of spots in both malignant and non-malignant clusters.* A dot represents a spot. Two groups were compared by calculating the Cohen's d effect size and two-sided Wilcoxon rank-sum test. Each group is also summarized as a violin plot smoothed by a kernel density function.

d, *Spatial distribution of various cell types across the tumor tissue*.

e, *Spatial localization of cell sublineages.* The cell type of a Slide-seq bead is defined by the most abundant cell type in this bead.

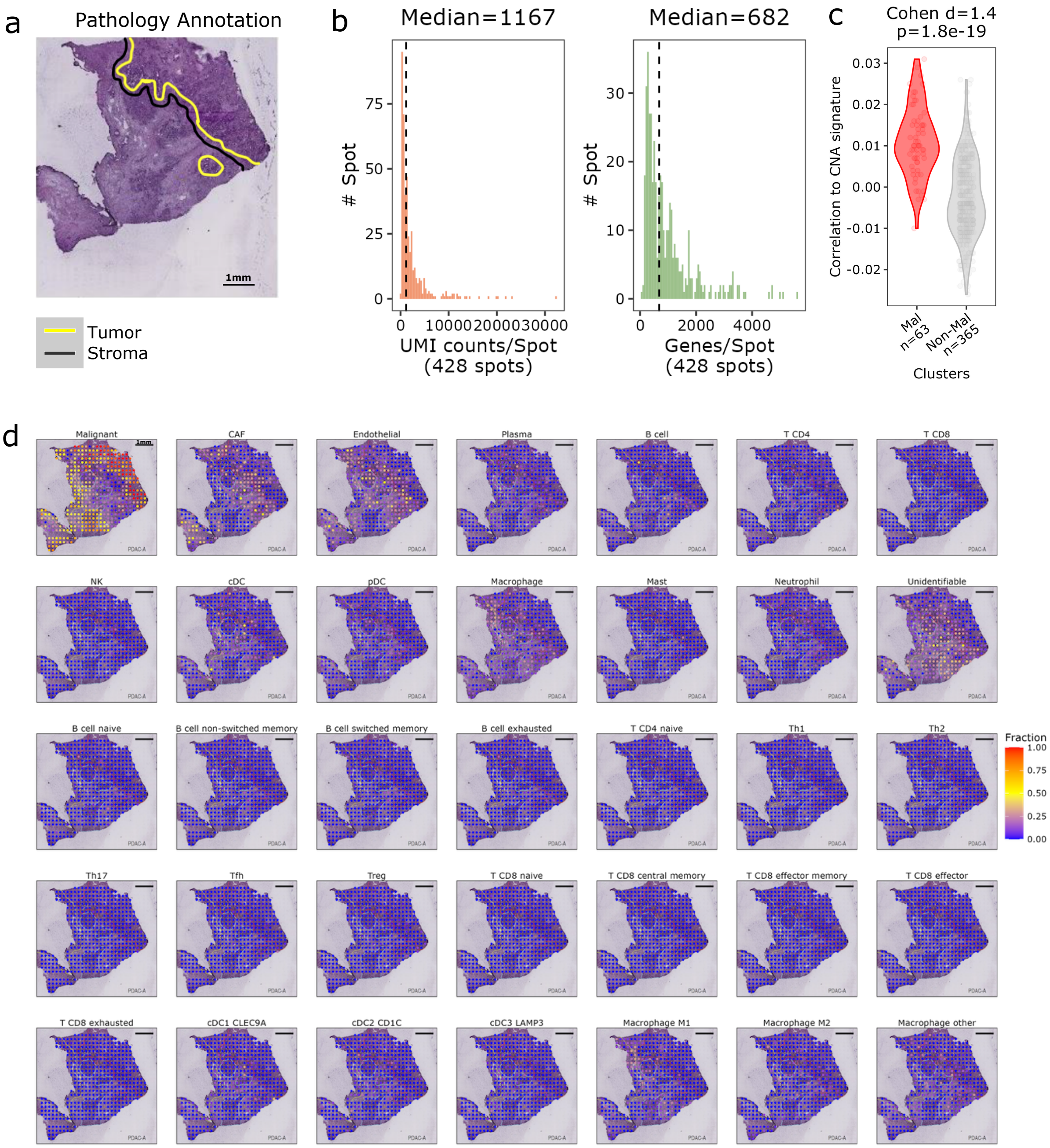

Supplementary Fig. 11. **SpaCET results of pancreatic ductal adenocarcinoma ST data**, related to Fig. 3.

a, *The H&E-stained tumor tissue image with pathology annotations.*

b, *Histogram of UMI counts and genes per spot.* The dashed line represents the median value across all spots.

c, *Pearson correlation between the cancer type-specific CNA signature and the gene expression of spots in both malignant and non-malignant clusters.* A dot represents a spot. Two groups were compared by calculating the Cohen's d effect size and two-sided Wilcoxon rank-sum test. Each group is also summarized as a violin plot smoothed by a kernel density function.

d, *Spatial distribution of various cell types across the tumor tissue*.

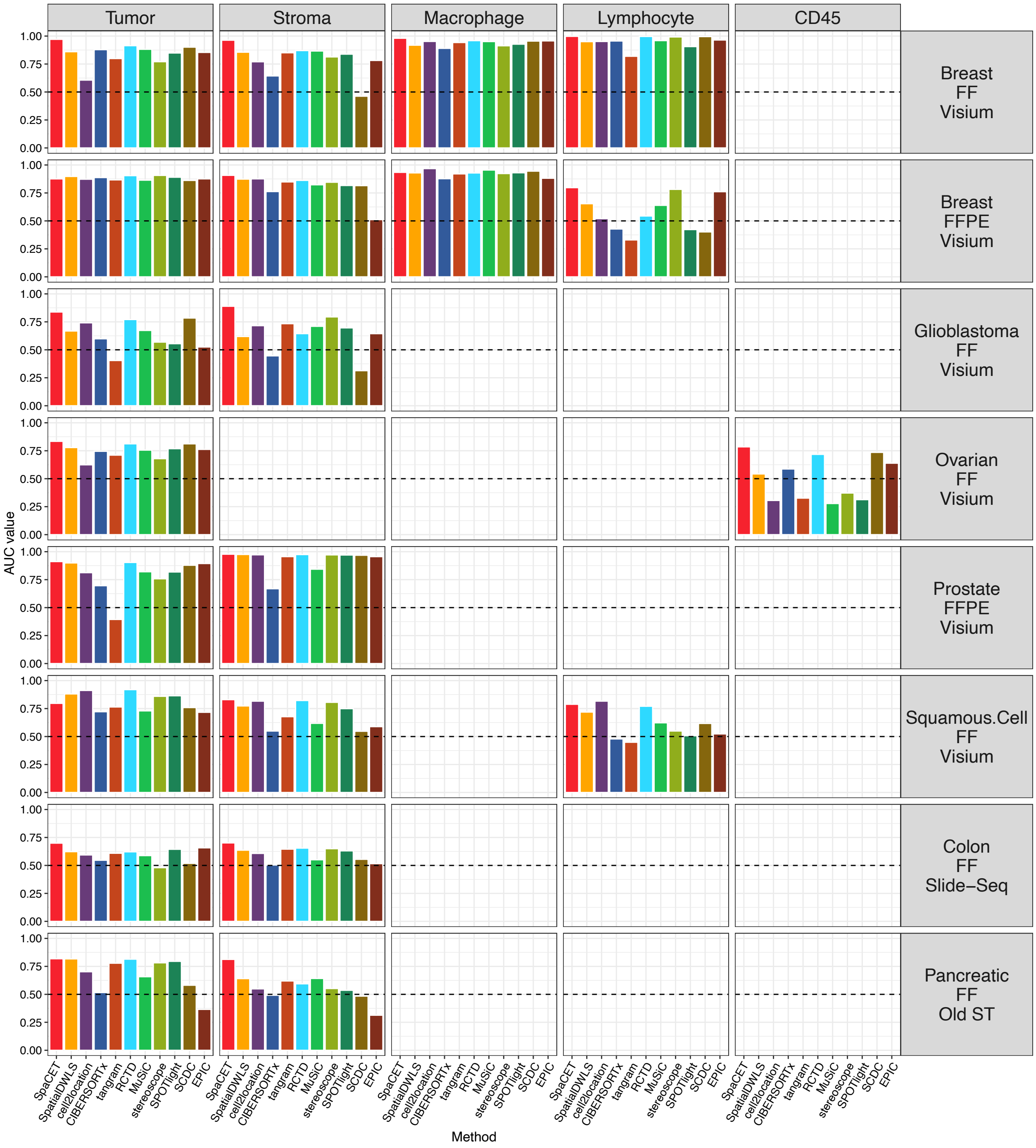

Supplementary Fig. 12. **Performance comparisons of eight ST datasets on seven cancer types**, related to Fig. 3 and Fig. 4

The AUC values of SpaCET and alternative methods for predicting different cell types (column) across ST datasets (row). The dashed line represents AUC = 0.5 as the random expectation. AUC: Area under the ROC Curve. ROC: Receiver Operating Characteristic; FF: Fresh frozen; FFPE: Formalin-fixed paraffin-embedded; ST: spatial transcriptomics.

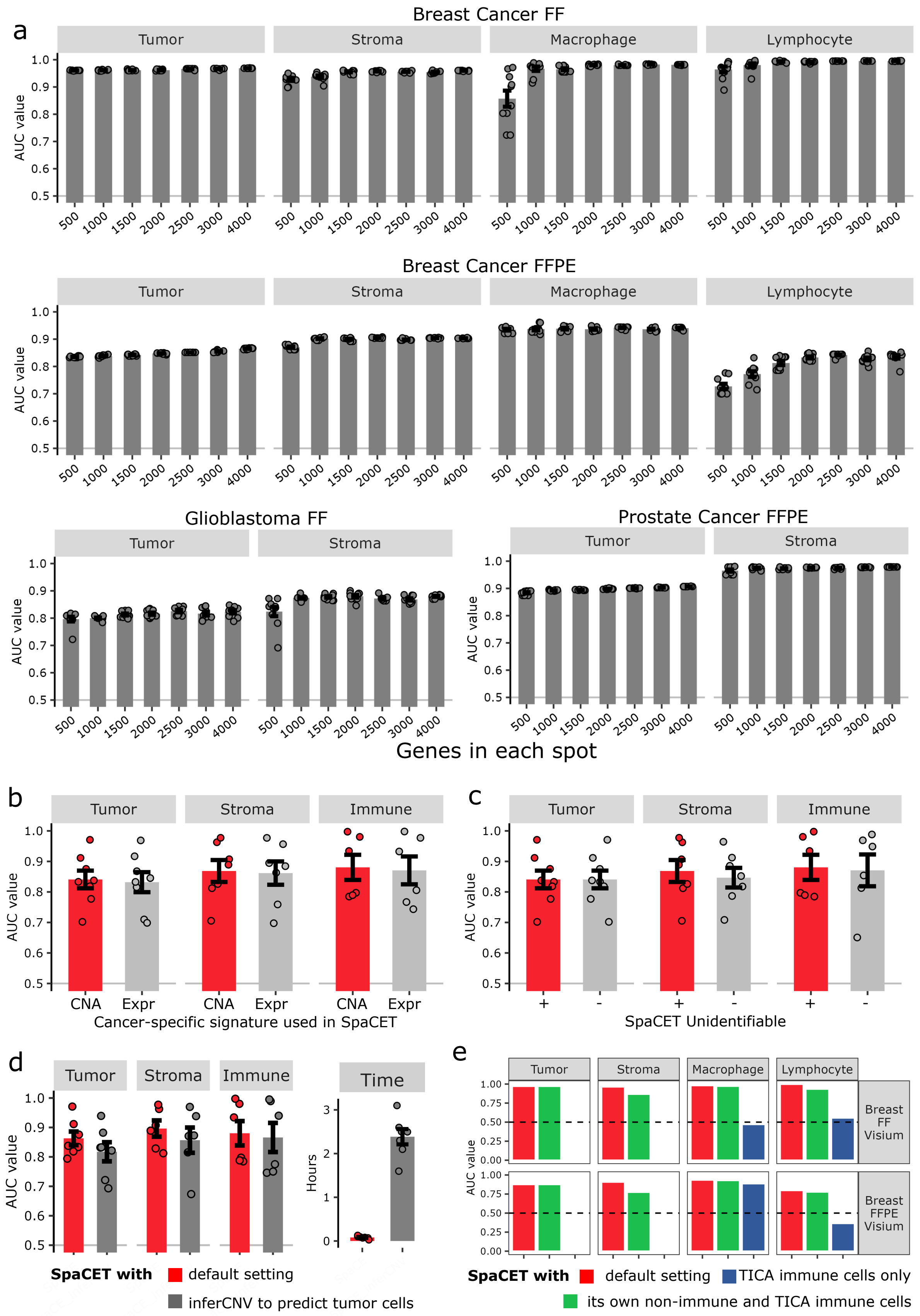

a
Breast Cancer FF
Tumor
Stroma
Macrophage
Lymphocyte
AUC value
Breast Cancer FFPE
Glioblastoma FF
Prostate Cancer FFPE
Genes in each spot
b
Tumor
Stroma
Immune
CNA
Expr
Cancer-specific signature used in SpaCET
c
SpaCET Unidentifiable
d
Time
Hours
SpaCET with
default setting
inferCNV to predict tumor cells
e
Breast FF Visium
Breast FFPE Visium
TICA immune cells only
its own non-immune and TICA immune cells

Supplementary Fig. 13. **Robustness and algorithm variations of SpaCET.** related to Fig. 3
a, *Deconvolution results of SpaCET on the randomly downsampled ST datasets.* The gene counts per spot were sampled from 4000 to 500 genes. Each dot represents a randomization replicate (10 dots in total). Bar height denotes the average AUC values across ten randomizations; error bars denote standard errors.
b, *Deconvolution results of SpaCET by using cancer type-specific CNA and expression signatures*. Each dot represents an ST dataset (n=8). The sub-panels represent the prediction of distinct regions from tumor tissue. The “immune” subpanel contains macrophage, lymphocyte, and CD45 shown in Supplementary Fig. 12. Bar height denotes the average AUC values across ST datasets; error bars denote standard errors.
c, *Deconvolution results of SpaCET with and without unidentifiable components*. Each dot represents an ST dataset (n=8). The annotations are the same as panel b.
d, *Deconvolution results of SpaCET with distinct malignant cell prediction methods.* Each dot represents an ST dataset (n=7). The red bar shows the output of SpaCET with default settings whereas the grey bar presents the results of the inferCNV-based strategy. Briefly, the inferCNV-based method predicted CNA values for genomics regions and genes from each ST transcriptomics data and determined the malignant cell abundance in each ST spot by the inferred CNA intensities. The annotations are the same as panel b.
e, *Deconvolution results of SpaCET with various references.* Based on the two breast cancer ST datasets, we did another two runs of SpaCET. One (green bar) is to replace the immune cells in our SpaCET atlas with a recently published tumor immune cell atlas (TICA). The other one (blue bar) is to directly decompose ST data by using TICA immune cells only, in which the deconvolution results only contain immune cells. The red bar shows the output of SpaCET with default settings.

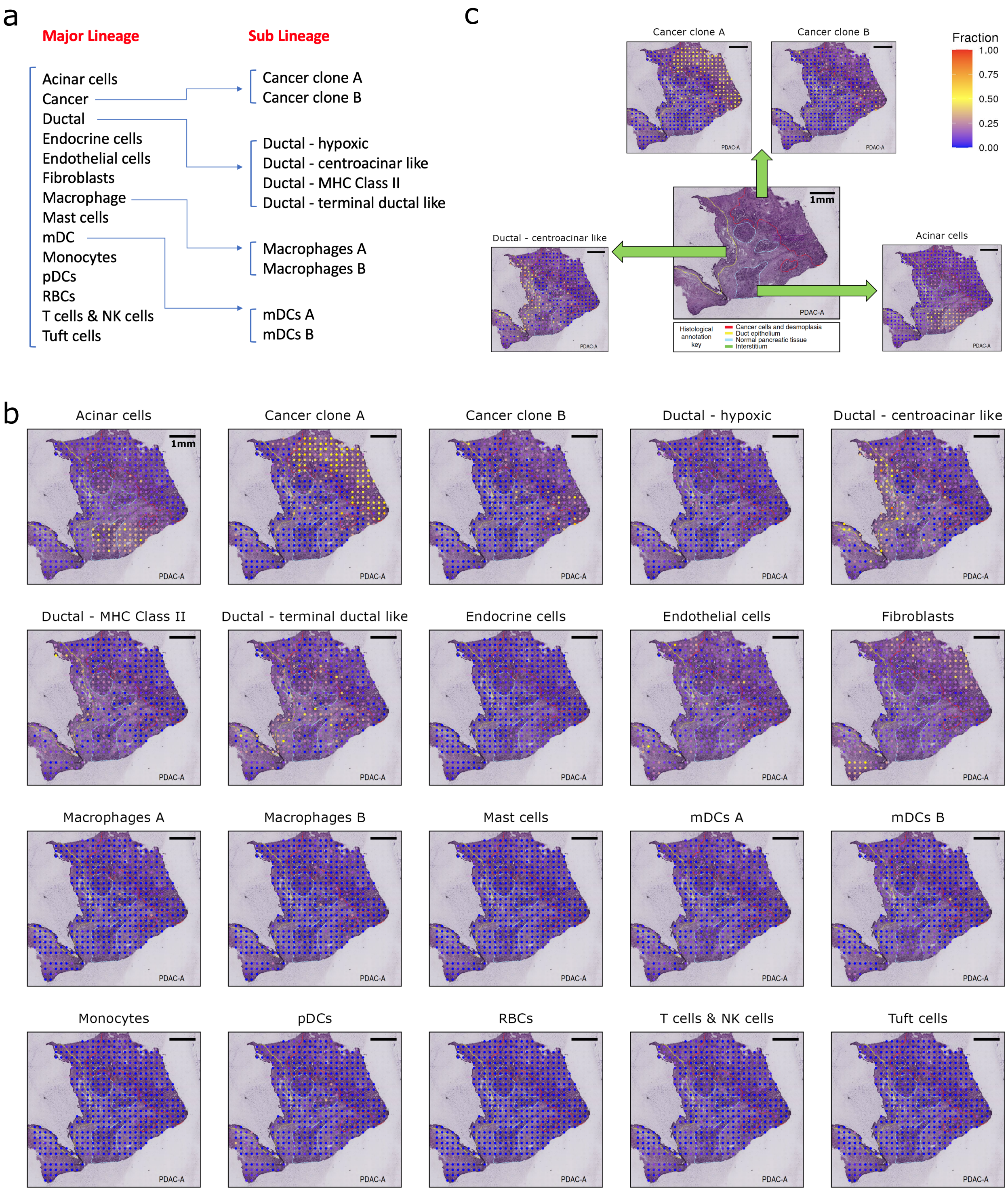

Supplementary Fig. 14. **SpaCET results of pancreatic ductal adenocarcinoma ST data with matched scRNA-seq dataset**.

a, *Hierarchical tree of cell types from the matched scRNA-seq data set.*

b, *Deconvolution results by SpaCET with the matched scRNA-seq as reference profiles.*

c, *Pathology annotations of the H&E-stained tissue image from the original publications, matched with SpaCET deconvolution results on relevant cell types.*

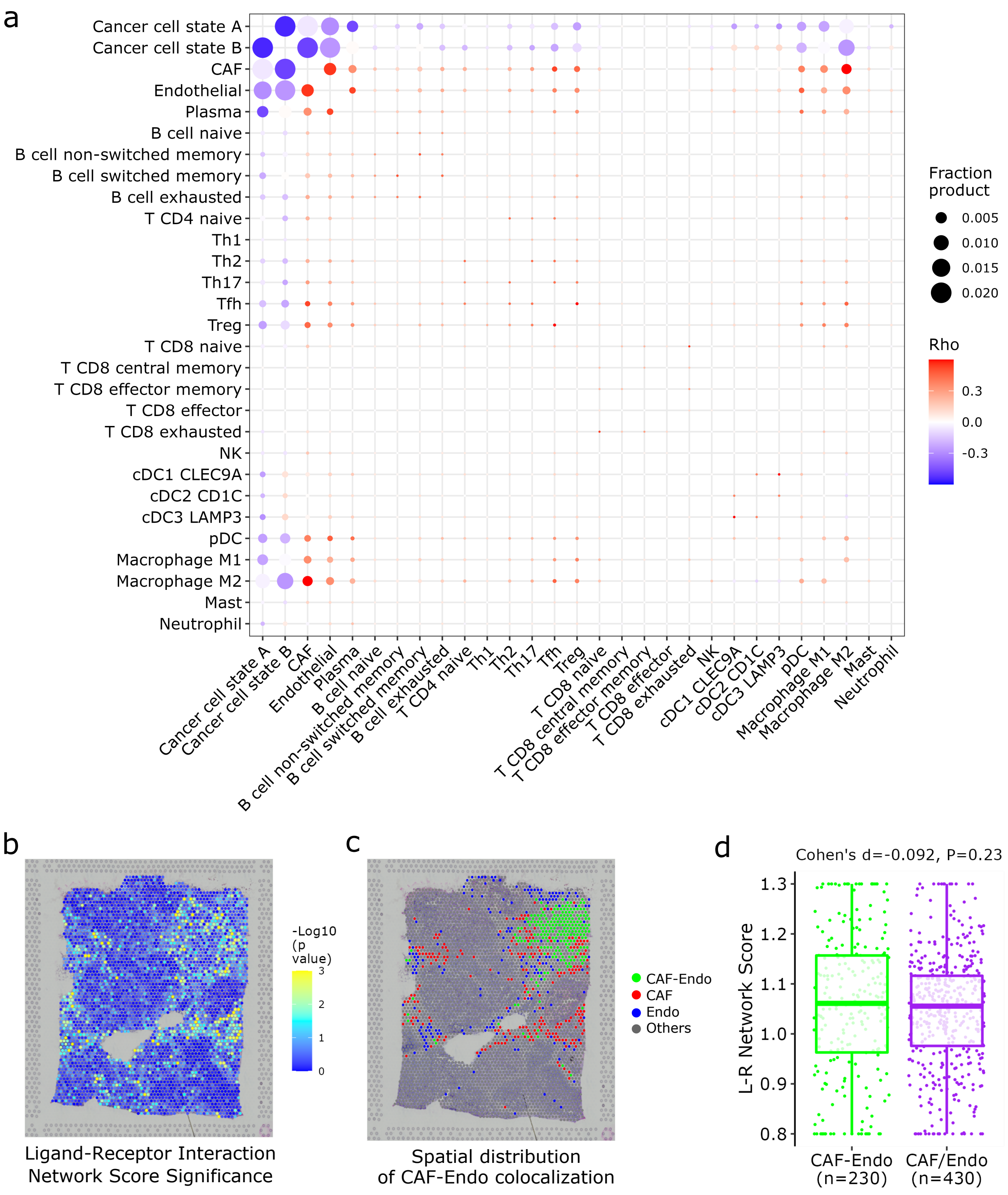

Supplementary Fig. 15. CAF-M2 interaction in a breast tumor, related to Fig. 5.
a, *Colocalization analysis between cell types as Spearman correlations (Rho)*. The size of a node refers to the product of average fractions of a cell-type pair across all spots.
b, *Ligand-Receptor (L-R) network score significance for all ST spots in the breast tumor.*

c, *Spatial distribution of CAF-Endothelial colocalized, CAF-dominated, and Endothelial-dominated spots.*
d, *Difference of L-R interaction network score between CAF-Endothelial colocalized spots and CAF/Endothelial-dominated spots in panel c*. The values of two groups were compared by using Cohen's d effect size and two-sided Wilcoxon rank-sum test. For the boxplot, the thick line represents the median value. The bottom and top of the boxes are the 25th and 75th percentiles (interquartile range). The whiskers encompass 1.5 times the interquartile range.

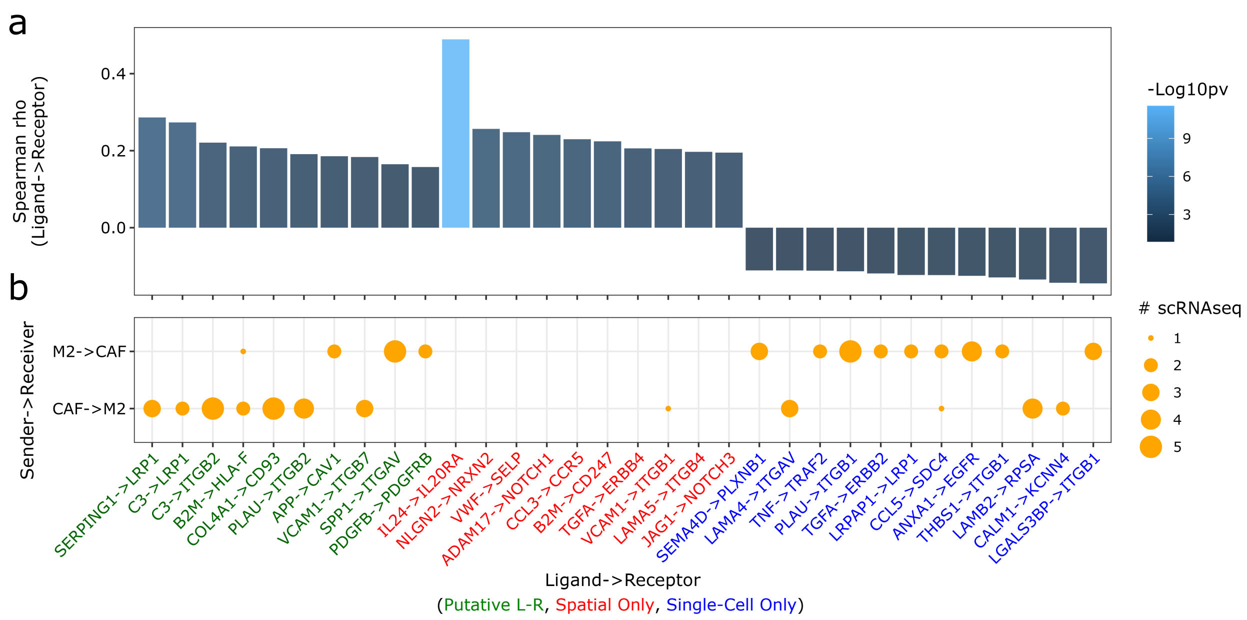

Supplementary Fig. 16. Ligand-Receptor interactions mediating CAF-M2 interactions, related to Fig. 6.

a, *Spearman correlation Rho between ligand and receptor expression across CAF-M2 colocalized spots of breast cancer ST data*.

b, *The number of scRNA-seq datasets that an L-R pair was estimated to be significant between CAF and M2 macrophage clusters in our single-cell data collection (*Supplementary Table 2*).* Because ST spot data contains the mixture transcriptome from a few cell types, thus cannot directly reveal the cell source of gene expression. We integrated scRNA-seq data to reveal the cell origin that expressed each ligand or receptor. The X-axis shows putative L-R pairs mediating CAF-M2 interaction and two types of false positives. Spatial-only false positive is the L-R interaction happening in spatial proximity but not in single-cell data analysis. These L-R interactions could happen between the same cell type but not between two cell types. Single-cell-only false positive is the L-R interaction derived from dissociated single-cell data analysis but not in the spatial proximity in the ST dataset analyzed.

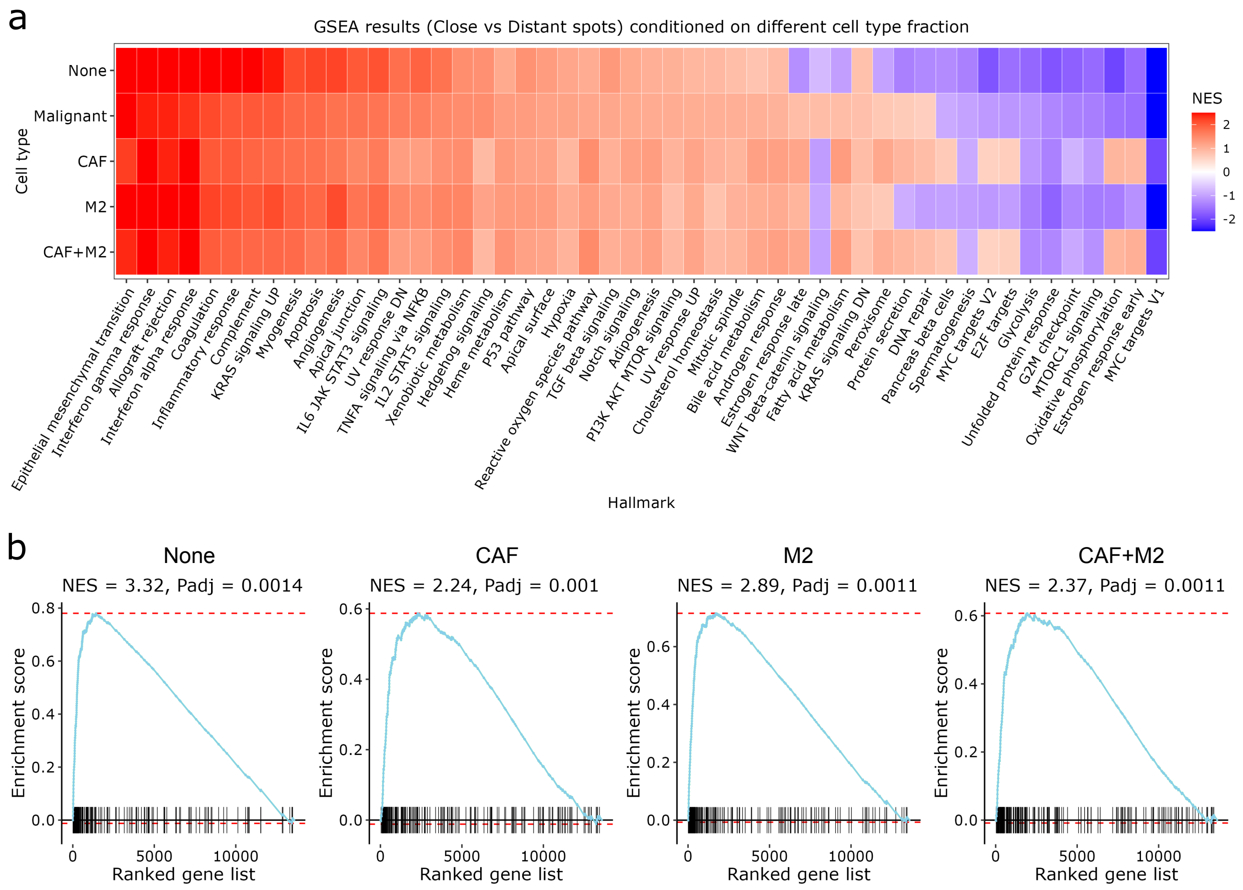

Supplementary Fig. 17. **Comparison between close and distant malignant cells to CAF-M2 interaction spots**, related to Fig. 6.

a, *Gene set enrichment analysis (GSEA) of pathways between close and distant malignant cell spots to CAF-M2 interaction spots conditioned on none, malignant, CAF, M2, and CAF+M2 cell fractions (Methods)*. In the y-axis, “None” means no control whereas “CAF+M2” means the sum cell fraction of CAF and M2. NES: normalized enrichment score.

b, *GSEA enrichment of epithelial-mesenchymal transition pathway (1st column) from panel a.* The GSEA plots conditioned on none, CAF, M2, and CAF+M2 cell fraction are shown in this panel whereas the GSEA plot conditioned on malignant cell fraction is shown in Fig. 6e. The P-value is computed through the two-sided permutation test (n = 1000 randomizations) adjusted by the Benjamini-Hochberg procedure.

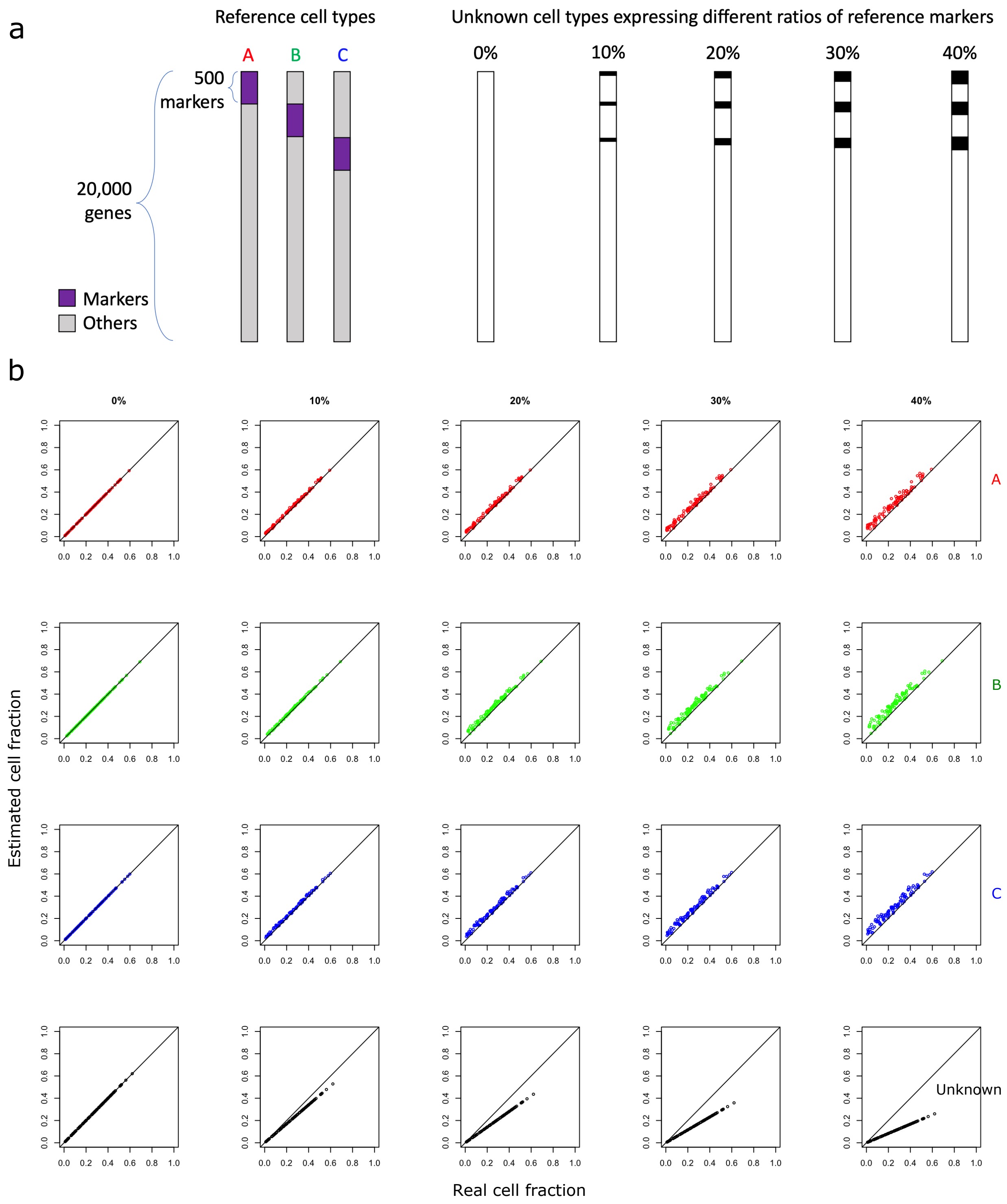

Supplementary Fig. 18. **Influence of reference markers expressed in unknown cell types on the SpaCET performance.**

a, *Simulation scheme of reference and unknown cell types.* Each of three reference cell types (i.e., A, B, and C) has 500 marker genes. We generated multiple unknown cell type profiles, expressing different ratios (0~40%) of reference markers. For example, 10% means that 10% of reference marker genes from cell types A, B, and C are expressed in the unknown cell type.

b, *Deconvolution results of simulated mixtures.* For each setting of unknown cell type, we mixed expression profiles of four cell types (i.e., A, B, C, and unknown) with random compositions to obtain 100 different mixtures. Then, SpaCET decomposes these mixtures by using cell-type A, B, and C as reference profiles. Each row represents the deconvolution results of a cell-type with different settings of unknown cell types.

Supplementary Tables

Supplementary Table 1. A gene pattern dictionary of copy number alterations (CNA) and tumor-normal expression differences generated from The Cancer Genome Atlas (TCGA). Certain tumor types do not have normal tissue controls (n < 10 patients); thus, we do not generate tumor-normal profiles for them.

Cancer	Full Name	CNA	Expr
ACC	Adrenocortical carcinoma	✓	
BLCA	Bladder Urothelial Carcinoma	✓	✓
BRCA	Breast invasive carcinoma	✓	✓
CESC	Cervical squamous cell carcinoma and endocervical adenocarcinoma	✓	
CHOL	Cholangiocarcinoma	✓	
COAD	Colon adenocarcinoma	✓	✓
ESCA	Esophageal carcinoma	✓	✓
GBM	Glioblastoma multiforme	✓	
HNSC	Head and Neck squamous cell carcinoma	✓	✓
KICH	Kidney Chromophobe	✓	✓
KIRC	Kidney renal clear cell carcinoma	✓	✓
KIRP	Kidney renal papillary cell carcinoma	✓	✓
LGG	Brain Lower Grade Glioma	✓	
LIHC	Liver hepatocellular carcinoma	✓	✓
LUAD	Lung adenocarcinoma	✓	✓
LUSC	Lung squamous cell carcinoma	✓	✓
MESO	Mesothelioma	✓	
OV	Ovarian serous cystadenocarcinoma	✓	
PAAD	Pancreatic adenocarcinoma	✓	
PCPG	Pheochromocytoma and Paraganglioma	✓	
PRAD	Prostate adenocarcinoma	✓	✓
READ	Rectum adenocarcinoma	✓	✓
SARC	Sarcoma	✓	
SKCM	Skin Cutaneous Melanoma	✓	
STAD	Stomach adenocarcinoma	✓	✓
TGCT	Testicular Germ Cell Tumors	✓	
THCA	Thyroid carcinoma	✓	✓
UCEC	Uterine Corpus Endometrial Carcinoma	✓	✓
UCS	Uterine Carcinosarcoma	✓	
UVM	Uveal Melanoma	✓	
PANCAN	Pan-cancer		✓

Supplementary Table 2. Tumor scRNA-seq datasets for simulation and reference generation.

GEO ID	Cancer Type	Platform	# Cell	# Patient	PubMed ID
E-MTAB-6149	Non-small cell lung cancer	10x	39,323	5	29988129
GSE103322	Head and neck squamous cell carcinoma	Smart-Seq2	5,902	21	29198524
GSE114725	Breast cancer	10x / InDrop	21,000	8	29961579
GSE115978	Melanoma	Smart-Seq2	7,186	31	30388455
GSE123139	Melanoma	MARS-seq	47,772	25	30595452
GSE127465	Non-small cell lung cancer	InDrop	40,362	7	30979687
GSE140228	Hepatocellular carcinoma	10x	18,621	5	31675496
GSE140228	Hepatocellular carcinoma	Smart-Seq2	2,423	6	31675496
GSE146771	Colorectal cancer	10x	10,694	10	32302573
GSE146771	Colorectal cancer	Smart-Seq2	5,220	10	32302573

Supplementary Table 3. Tumor spatial transcriptomics datasets used in this study. FF: Fresh frozen; FFPE: Formalin-fixed paraffin-embedded; H&E: Hematoxylin & Eosin. *The early in situ capturing method from which 10x Visium was developed.

GEO ID	Cancer Type	Platform	# Spot	# Gene	Preservation	Staining	Source
N/A	Breast cancer	Visium	3,813	22,953	FF	H&E	10x Genomics
N/A	Breast cancer	Visium	2,518	17,649	FFPE	H&E	10x Genomics
N/A	Glioblastoma	Visium	3,468	25,275	FF	H&E	10x Genomics
N/A	Ovarian cancer	Visium	3,493	24,012	FF	DAPI,Anti-CD45, Anti-PanCK	10x Genomics
N/A	Prostate cancer	Visium	4,371	16,905	FFPE	H&E	10x Genomics
GSE144240	Squamous cell carcinoma	Visium	3,650	20,255	FF	H&E	PMID: 32579974
SCP1278	Colon cancer	Slide-seq	18,288	16,270	FF	H&E	PMID: 34912115
GSE111672	Pancreatic ductal adenocarcinoma	The early in situ capturing method*	428	14,574	FF	H&E	PMID: 31932730

Supplementary Table 4. *Estimated cell-cell interactions based on the decomposed cell type fractions from different deconvolution methods*.

Methods	CAF-M2	CAF-Endothelial
SpaCET	✓	x
SpatialDWLS	✓	✓
cell2location	✓	x
CIBERSORTx	✓	✓
tangram	✓	✓
RCTD	✓	✓
MuSiC	✓	✓
stereoscope	✓	✓
SPOTlight	✓	✓
SCDC	✓	✓
EPIC	✓	✓
