# Supplementary material for: Estimation of cell lineages in tumors from spatial transcriptomics data
Source: Nat Commun. 2023 Feb 2;14:568. doi: 10.1038/s41467-023-36062-6 (PMC9895078; doi:10.1038/s41467-023-36062-6)
Supplement: Supplementary file 3 — Reporting Summary [file 41467_2023_36062_MOESM3_ESM.pdf]

nature portfolio

Corresponding author(s): Peng Jiang

Last updated by author(s): Dec 18, 2022

Reporting Summary

Nature Portfolio wishes to improve the reproducibility of the work that we publish. This form provides structure for consistency and transparency in reporting. For further information on Nature Portfolio policies, see our Editorial Policies and the Editorial Policy Checklist.

Statistics

For all statistical analyses, confirm that the following items are present in the figure legend, table legend, main text, or Methods section.

n/a	Confirmed	
☐	☒	The exact sample size (*n*) for each experimental group/condition, given as a discrete number and unit of measurement
☐	☒	A statement on whether measurements were taken from distinct samples or whether the same sample was measured repeatedly
☐	☒	The statistical test(s) used AND whether they are one- or two-sided<br>*Only common tests should be described solely by name; describe more complex techniques in the Methods section.*
☒	☐	A description of all covariates tested
☐	☒	A description of any assumptions or corrections, such as tests of normality and adjustment for multiple comparisons
☐	☒	A full description of the statistical parameters including central tendency (e.g. means) or other basic estimates (e.g. regression coefficient) AND variation (e.g. standard deviation) or associated estimates of uncertainty (e.g. confidence intervals)
☐	☒	For null hypothesis testing, the test statistic (e.g. *F*, *t*, *r*) with confidence intervals, effect sizes, degrees of freedom and *P* value noted<br>*Give P values as exact values whenever suitable.*
☒	☐	For Bayesian analysis, information on the choice of priors and Markov chain Monte Carlo settings
☐	☒	For hierarchical and complex designs, identification of the appropriate level for tests and full reporting of outcomes
☐	☒	Estimates of effect sizes (e.g. Cohen's *d*, Pearson's *r*), indicating how they were calculated

Our web collection on statistics for biologists contains articles on many of the points above.

Software and code

Policy information about availability of computer code

Data collection	No Software was used.
Data analysis	The SpaCET R package version 1.0.0 was used to analyze the data. The source code is available at https://github.com/data2intelligence/SpaCET. Alternative deconvolution software for comparative analysis include stereoscope 0.2.0, SPOTlight 0.1.7, Tangram 1.0.0, RCTD 1.2.0, SpatialDWLS from Giotto 1.1.1, cell2location 0.6a0, EPIC 1.1.5, CIBERSORTx 1.0, MuSiC 0.2.0, and SCDC 0.0.0.9000. Other R packages used in our analysis include BiRewire 3.26.5, inferCNV 1.10.1, limma 3.50.1, SingleR 1.8.1, and fgsea 1.20.0.

For manuscripts utilizing custom algorithms or software that are central to the research but not yet described in published literature, software must be made available to editors and reviewers. We strongly encourage code deposition in a community repository (e.g. GitHub). See the Nature Portfolio guidelines for submitting code & software for further information.

Data

Policy information about availability of data

All manuscripts must include a data availability statement. This statement should provide the following information, where applicable:

- Accession codes, unique identifiers, or web links for publicly available datasets
- A description of any restrictions on data availability
- For clinical datasets or third party data, please ensure that the statement adheres to our policy

Ten tumor single-cell RNA-seq datasets were collected for simulation and reference generation, i.e., one from ArrayExpress (https://www.ebi.ac.uk/arrayexpress/

experiments/E-MTAB-6149/) and nine from GEO (GSE103322, GSE114725, GSE115978, GSE123139, GSE127465, GSE140228, and GSE146771).
Among the eight real spatial transcriptomics datasets, five were collected from the 10x Genomics (https://support.10xgenomics.com/spatial-gene-expression/datasets), two from GEO (GSE144240, GSE111672), and one from SCP (https://singlecell.broadinstitute.org/single_cell/study/SCP1278).
More details on both single-cell RNA-seq and spatial transcriptomics datasets are listed in Supplementary Table 2 and 3, respectively. The Cancer Genome Atlas (TCGA) data are available for download at https://gdc.cancer.gov/. Hallmark gene sets (v.7.2) from MSigDB are available at https://www.gsea-msigdb.org. Source data are provided as a Source Data file.

Human research participants

Policy information about studies involving human research participants and Sex and Gender in Research.

Reporting on sex and gender	Not relevant because we used public data sets.
Population characteristics	N/A
Recruitment	N/A
Ethics oversight	N/A

Note that full information on the approval of the study protocol must also be provided in the manuscript.

Field-specific reporting

Please select the one below that is the best fit for your research. If you are not sure, read the appropriate sections before making your selection.

☒ Life sciences ☐ Behavioural & social sciences ☐ Ecological, evolutionary & environmental sciences

For a reference copy of the document with all sections, see nature.com/documents/nr-reporting-summary-flat.pdf

Life sciences study design

All studies must disclose on these points even when the disclosure is negative.

Sample size	SpaCET was evaluated by using simulation and real spatial transcriptomics (ST) data. For the simulation analysis, we collected ten cancer single cell RNA-seq datasets to synthesize ST data. For real ST data, we also collected eight cancer ST datasets on seven tumor types. The number of datasets in this study is beyond the published studies for other deconvolution tools.
Data exclusions	For each ST data set, we used all spots that were detected to have non-zero expression as determined by their original data source. No data were excluded from this study.
Replication	For assessing the robustness of SpaCET with respect to the gene coverage of ST data, we repeated our analysis 10 times for each down-sampling from 4000 to 500 genes per spot. All attempts at replication were successful.<br>All other simulated and real ST datasets were analyzed once because they do not have replications.
Randomization	Randomization was not relevant because the ST datasets collected from public databases were analyzed separately.
Blinding	Our pathologist performed double-blind annotation of tumor and immune cell regions in the H&E images paired with ST data, without knowing our computational results.<br>Blinding was not relevant to other analyses because our results were based on objective computation results instead of human derived labels.

Reporting for specific materials, systems and methods

We require information from authors about some types of materials, experimental systems and methods used in many studies. Here, indicate whether each material, system or method listed is relevant to your study. If you are not sure if a list item applies to your research, read the appropriate section before selecting a response.

Materials & experimental systems

n/a	Involved in the study
☒	☐ Antibodies
☒	☐ Eukaryotic cell lines
☒	☐ Palaeontology and archaeology
☒	☐ Animals and other organisms
☒	☐ Clinical data
☒	☐ Dual use research of concern

Methods

n/a	Involved in the study
☒	☐ ChIP-seq
☒	☐ Flow cytometry
☒	☐ MRI-based neuroimaging
